# COVID-19 in Germany and China: mitigation versus elimination strategy

**DOI:** 10.1080/16549716.2021.1875601

**Published:** 2021-01-20

**Authors:** Guangyu Lu, Oliver Razum, Albrecht Jahn, Yuying Zhang, Brett Sutton, Devi Sridhar, Koya Ariyoshi, Lorenz von Seidlein, Olaf Müller

**Affiliations:** a Department of Public Health, Medical College, Yangzhou University, Yangzhou, China; b Department of Epidemiology & International Public Health, School of Public Health, Bielefeld University, Bielefeld, Germany; c Institute of Global Health, Medical School, Ruprecht-Karls-University Heidelberg, Heidelberg, Germany; d Ministry of Health, Melbourne, Australia; e Global Health Governance Programme, Usher Institute of Population Health Sciences and Informatics, University of Edinburgh, Edinburgh, UK; f School of Tropical Medicine and Global Health, Nagasaki University, Nagasaki, Japan; g Mahidol Oxford Tropical Medicine Research Unit, Faculty of Tropical Medicine, Mahidol University, Bangkok, Thailand

**Keywords:** COVID-19, pandemic, China, Germany, health policy

## Abstract

**Background**: The COVID-19 pandemic shows variable dynamics in WHO Regions, with lowest disease burden in the Western-Pacific Region. While China has been able to rapidly eliminate transmission of SARS-CoV-2, Germany – as well as most of Europe and the Americas – is struggling with high numbers of cases and deaths.

**Objective**: We analyse COVID-19 epidemiology and control strategies in China and in Germany, two countries which have chosen profoundly different approaches to deal with the epidemic.

**Methods**: In this narrative review, we searched the literature from 1 December 2019, to 4 December 2020.

**Results**: China and several neighbours (e.g. Australia, Japan, South Korea, New Zealand, Thailand) have achieved COVID-19 elimination or sustained low case numbers. This can be attributed to: (1) experience with previous coronavirus outbreaks; (2) classification of SARS-CoV-2 in the highest risk category and consequent early employment of aggressive control measures; (3) mandatory isolation of cases and contacts in institutions; (4) broad employment of modern contact tracking technology; (5) travel restrictions to prevent SARS-CoV-2 re-importation; (6) cohesive communities with varying levels of social control.

**Conclusions**: Early implementation of intense and sustained control measures is key to achieving a near normal social and economic life.

## Background

Following the severe acute respiratory syndrome corona virus 1 (SARS-CoV-1) outbreak in 2002 in China and the Middle East respiratory syndrome coronavirus (MERS-CoV) outbreak in 2012 in Jordan, the occurrence of coronavirus disease 2019 (COVID-19) caused by the severe acute respiratory syndrome virus 2 (SARS-CoV-2) is now the third outbreak of a highly pathogenic zoonotic coronavirus disease in the 21^st^ century [1,2]. SARS-CoV-2 was first reported in December 2019 in Wuhan, China, and has rapidly spread globally since [3]. On 31 January 2020, the outbreak was declared a *Public Health Emergency of International Concern* and on 11 March 2020, a global pandemic by the World Health Organization (WHO) [4]. By mid-December, 2020, there are more than 70 million reported COVID-19 cases (not all of them symptomatic), and about 1.6 million reported deaths world-wide [5]. Case fatality rates (CFRs) and infection fatality rates (IFRs) of COVID-19 vary over time both locally and globally, depending on a number of factors such as the intensity of testing in respective countries, the age distribution of affected populations, the proportion of risk groups (elderly persons, persons with chronic diseases), and the availability, accessibility, and quality of health services [6,7]. While CFR estimates are usually higher compared to IFRs as they are simply calculated from the number of positive tests without consideration of the true denominator, IFR estimates are producing a more realistic picture based on available data from closed cohort studies and serological surveys; they range from 0.2% to 0.4% globally and from 0.4 to 1% in Germany [8,9].

SARS-CoV-2 is mainly transmitted through droplets and aerosols during conversations, shouting, singing, and exercising, and where people congregate in poorly ventilated indoor places; transmission through fomites seems to play a minor role [10–12]. The virus initially replicates in the upper respiratory tract and COVID-19 cases already become infectious the days before symptoms occur; this is a critical difference to the epidemiological characteristics of SARS-CoV-1 in which infected persons start infecting others only after the onset of symptoms; this has major implications regarding the success of control measures [13].

Health workers are now better protected compared to the beginning of the pandemic but remain a major risk group [7]. Today, most transmission occurs where unprotected people stay closely together for prolonged periods of time, as it is primarily the case in households and during informal gatherings of relatives and friends [11–14]. Transmission risk is also high in crowded places such as dormitories, nursing homes, prisons, refugee camps, cruise ships, and certain work places [11,12]. Superspreading, which is associated with large public or private gatherings (e.g. in churches or during community events), continues being responsible for large outbreaks [11].

**Figure 1.**
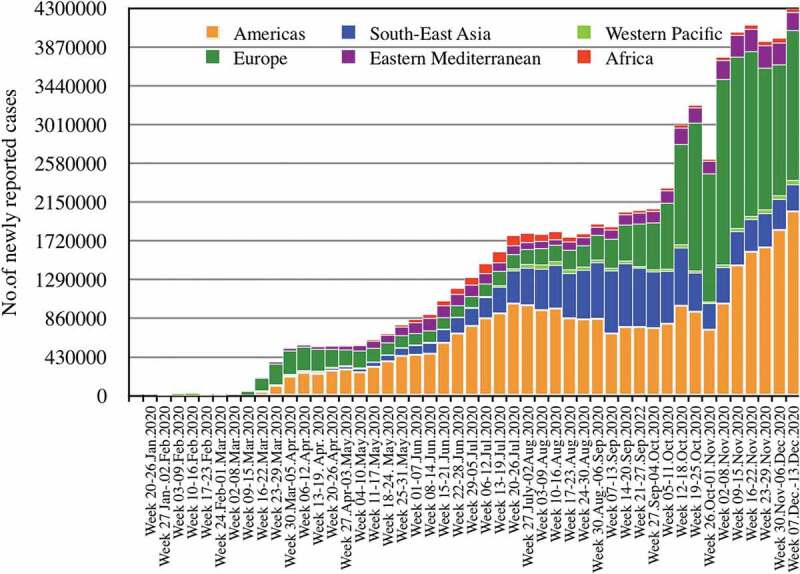
Number of COVID-19 cases reported weekly by WHO Region, 30 December 2019 through 13 December 2020

**Figure 2.**
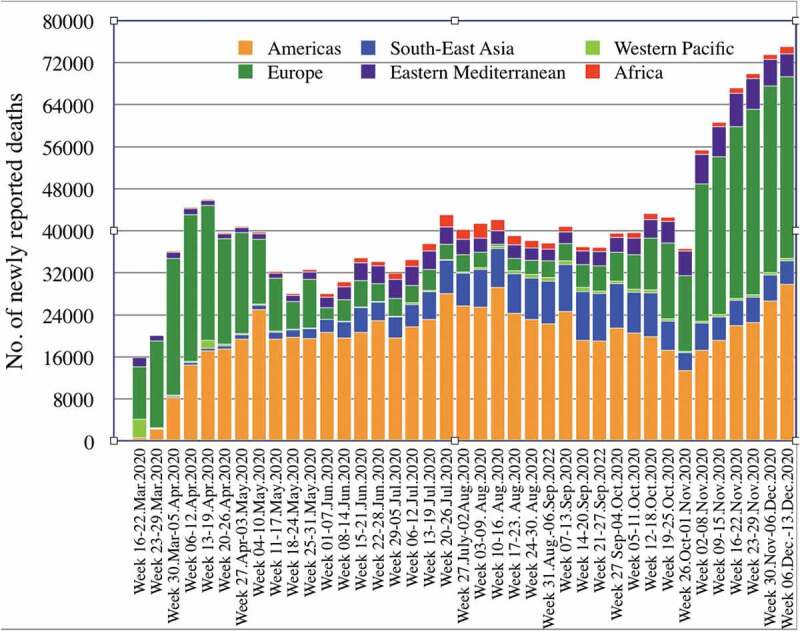
Number of COVID-19 deaths reported weekly by WHO Region, 30 December 2019 through 13 December 2020

The COVID-19 epidemics show variable dynamics in the different WHO Regions, with the Region of the Americas, the European Region and the South-East Asian Region carrying the highest burden of infections and deaths so far (Figures 1, 2, Table 1) [15]. The WHO European Region has experienced its first epidemic wave in March and April 2020, which – after painful lockdowns in most countries – appeared to be controlled during the following summer months [16]. In contrast, the WHO American Region has been facing a nearly uninterrupted and steadily increasing wave since March [5]. Starting with an unexpected rapid increase of new infections in autumn, Europe is now in the middle of a large second wave [16]. The WHO South-East Asia Region has been highly affected since March already, and India is reporting the second highest number of confirmed cases after the USA [5]. In contrast, countries in the WHO Eastern Mediterranean (with the exception of parts of the Middle East), the WHO Africa and the WHO Western Pacific Region have so far reported a much lower number of cases and deaths, with the countries in the Western Pacific Region reporting remarkably low numbers [15,17].

**Table 1.** Cumulative number of COVID-19 cases and deaths and proportion of global cases/deaths by WHO Region, as of 13 December 2020

WHO Region	Cumulative cases (%)	Cumulative deaths (%)
Americas	30,116,395 (43%)	776,708 (49%)
Europe	21,925,389 (31%)	484,570 (30%)
South-East Asia	11,361,437 (16%)	172,858 (11%)
Eastern Mediterranean	4,490,755 (6%)	111,635 (7%)
Africa	1,622,096 (2%)	35,879 (2%)
Western Pacific	960,020 (1%)	18,259 (1%)
**Global**	**70,476,836 (100%)**	**1,599,922 (100%)**

Different strategies have been proposed and tried to contain COVID-19 epidemics. Approaches range from reducing the incidence in a country to zero (‘aggressive suppression’, meaning an elimination strategy) or at least to very low levels so that nearly all infections can be identified and controlled through rapid testing, backward and forward tracking of people infected, and tracing their contacts (TTT) interventions (containment strategy), to keeping the incidence below the capacity limit of hospitals and intensive care units (‘flattening the curve’, meaning a mitigation strategy), to only protecting population groups at high risk for severe disease and deaths from SARS-CoV-2 infections (herd immunity strategy). All these strategies have pros and cons. Elimination and containment strategies need aggressive control measures, supported by the appropriate technology (e.g. tracing apps, mobile phone support, extensive testing) and strong political support; they have certain implications on individual data protection and personal liberties but can keep rates of SARS-CoV-2 infections, COVID-19 cases and deaths very low, thus presumably minimising long-term restrictions on personal liberties of societies [18,19]. Mitigation strategies aim to keep infection numbers low through moderate control measures, but usually have to be amended by lockdowns as soon as increasing SARS-CoV-2 infections risk to overwhelm the capacity of health services; thus, personal liberties are initially not compromised but this changes during lockdown times. Most importantly, this strategy is associated with significant morbidity and mortality [7,17]. Herd immunity strategies keep societal life largely normal but interfere with personal liberties and the right for health and life of risk groups; moreover, they accept high numbers of severe disease cases (including in younger age groups), a large excess mortality, and risk recurrent epidemics [7,17].

While elimination strategies have been implemented so far in only a handful of places such as China, various small islands and in New Zealand, containment strategies including intense behaviour change interventions have become successfully established in a number of other countries and territories of the WHO Western Pacific Region (e.g. Taiwan, Thailand, Vietnam, Australia, South Korea, Singapore) [7,20–22]. The vast majority of countries in the world has, however, implemented mitigation strategies, using various degrees of non-pharmaceutical interventions (NPI) (e.g. face masking, physical distancing rules, restrictions of movement and social gatherings) combined with TTT interventions as well as repeated lockdowns (movement restrictions for populations of regions or whole nations). Finally, implementing a herd immunity strategy in an industrialized country has only been tried in Sweden in a planned way, but might be the reality in many very low-income countries as well as in parts of India and Brazil [23,24].

In this paper, we present an analysis of the COVID-19 epidemiology in China and in Germany, two countries which have chosen profoundly different approaches to deal with the epidemic. While China has employed an elimination strategy from the beginning, Germany has also acted early but employed a mitigation strategy. The situation in China and Germany is also discussed in view of epidemiological developments in neighboring countries of the WHO Western Pacific and the WHO European Region.

## Methods

### Search strategy and selection criteria

Relevant websites of governmental agencies were searched, including the Chinese Centre for Disease Control (http://www.chinacdc.cn/), National Health Commission of the People’s Republic of China (http://www.nhc.gov.cn/xcs/yqtb/list_gzbd.shtml), and Germany’s Robert Koch Institute (http://www.rki.de), as well as PubMed, Google Scholar and preprint repositories (medRxiv and arXiv). The search was restricted to the period of 1 December 2019, to 4 December 2020. Only papers published in English, Chinese and German were reviewed. The search terms used were: ‘COVID-19ʹ or ‘SARS-CoV-2ʹ; and ‘China’ or ‘Germany’; and ‘heath policy’, or ‘response’, or ‘elimination’ or ‘containment’ or ‘suppression’ in the title and/or abstract of the article. We identified a total of 2,236 articles in the databases, of which 2,143 were excluded as duplicates or not relevant to the research question. The final reference list was generated on the basis of originality and relevance to the broad scope of this review. We included a total of 73 articles which referred to COVID-19 responses at a public health level and also described public health policies controlling or suppressing the COVID-19 epidemic. The article was presented in accordance with the Narrative Review Reporting Checklist (available at https://www.equator-network.org/reporting-guidelines/rameses-publication-standards-meta-narrative-reviews/).

## Results

### Epidemic development and response in China

In the initial weeks after the outbreak in Wuhan, China experienced an exponential growth of confirmed COVID-19 cases [7]. The Chinese authorities reacted rapidly and implemented large-scale public health measures, comprising a combination of a range of social distancing and other established NPIs [25–28]. Community workers and volunteers were key to implementation of the control measures, which were partly supported by use of big data [29–32]. The Chinese multi-sectoral response has been overseen from the beginning by the highest political level [33,34]. By the end of January, intense lockdowns were implemented first in Wuhan, then in the Hubei Province and finally in nearly all of China [7,31]. The lockdowns were accompanied by a very rapid construction of new hospitals in Wuhan, the re-allocation of several thousand medical staff from other parts of China to Hubei Province, intense surveillance and isolation/quarantine measures, and by severe travel restrictions in the whole country [7,25]. To avoid transmission of SARS-CoV-2 in households, isolation of mild and moderate COVID-19 cases took place in *Fangcang shelter hospitals*; these were large-scale, temporary hospitals, rapidly built by converting existing public venues, such as stadiums and exhibition centres, into health-care facilities [35]. In addition, the national government has established a comprehensive social security system to mitigate the suffering of Chinese society in the mid- and post-crisis periods [36].

**Figure 3.**
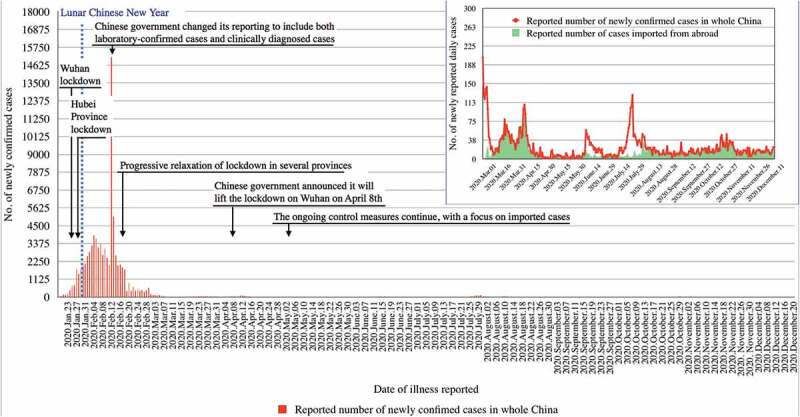
COVID-19 control interventions in China and number of laboratory-confirmed cases from 20 January to 20 December 2020

The stringent lockdown in Wuhan, which lasted from 23 January until 8 April 2020, appeared to have eliminated COVID-19; a city-wide mass PCR screening program in May 2020 has found only 300 asymptomatic and non-infectious positive cases among 10 million population tested [37]. Moreover, the number of reported COVID-19 cases declined substantially within a few weeks after the implementation of lockdowns in the whole of China, and the number of new autochthonous infections approached zero in early March 2020 (Figure 3) [7].

After transmission was under control, the intensity of public health interventions has been progressively relaxed in Chinese provinces. Nearly all newly confirmed COVID-19 cases in China have been imported cases since (Figure 3) [38]. A number of small outbreaks in a few cities were rapidly controlled through intense public health measures. The latest outbreaks took place in the cities of Kashgar (Xinjiang Province) and in Qingdao (Shandong Province) in October 2020; they were controlled by aggressive public health measures including testing of some 5 million and 10 million population, respectively, within a few days [39,40]. SARS-CoV-2 surveillance and COVID-19 control measures continue in China through intensive TTT measures. Figure 3 shows the epidemic curve of China with relevant epidemiological and control data. By December 21, China had reported a total of 95,135 confirmed cases and a total of 4,764 deaths (CFR = 5.0%) [5].

The COVID-19 epidemic in Hong Kong was characterized by several waves of imported cases followed by limited local transmissions [41,42]. The city authorities had applied immediate, strong and successful containment measures, including an aggressive escalation of border control, and a public health response facilitated by the experience with the SARS-CoV-1 outbreak in 2003 [43]. The implementation of early and intense NPIs has allowed Hong Kong to keep SARS-CoV-2 infections at very low levels without undertaking lockdowns [44].

In Taiwan, the COVID-19 epidemic was well controlled from the beginning [22,45]. Immediately after the first SARS outbreak, the Taiwanese government had established the National Health Command Centre, which coordinates the public health response to large infectious disease outbreaks [46]. All travellers from mainland China were quarantined at home and tracked through their mobile phone to ensure that they remained at home during the incubation period. Afterwards, Taiwan has been able to minimize COVID-19 cases without a lockdown due to proactively and rapidly responding to the pandemic, including border control, case identification, and quarantine of suspected cases [22,46]. Until December 21, there were only 766 reported cases with 7 deaths since the beginning of the pandemic (CFR = 0.9%) in a population of 24 million [5].

### Epidemic development and response in Germany

The first cases of COVID-19 were imported from China to southern Germany at the end of January 2020 [47,48]. These cases and their contacts were rapidly tracked down by the health system and transmission interrupted. By March 2020 the number of COVID-19 cases had started to increase exponentially [7]. Compared to highly affected European countries (e.g. Italy, Spain, France, UK), a lower CFR was observed in Germany during this first wave [49–52]. This has largely been explained by an early and broad testing strategy, an initially rather young SARS-CoV-2-infected population due to transmission mainly at hotspots such as carnival meetings and ski resorts, and a well-functioning healthcare system [7,53].

**Figure 4.**
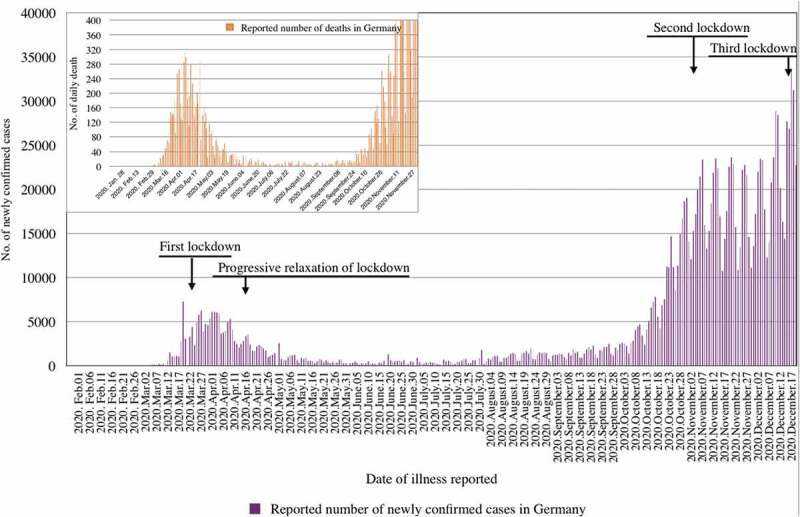
Covid-19 control interventions in Germany and number of laboratory-confirmed cases and deaths from 28 January to 20 December 2020

In view of increasing case numbers and severe consequences of the epidemic in neighbouring countries (in particular Italy), Germany implemented a lockdown from 22 March until 3 May 2020, which included closure of all non-essential business, preschools, schools, and much reduced activities in universities, as well as a ban of private and public gatherings, but no major restriction on individual movement [7,54]. The measures taken by the German government were largely accepted by the population, the number of cases declined steadily and stayed low during the summer months (Figure 3). Other factors likely to have contributed include an intense broad and science-oriented information and discussion of national and international epidemic developments in the German media, and an active and unified political leadership [55]. The warm and dry weather during summer, which allowed for outdoor social activities, probably contributed to keeping case load low [16,56]. At the end of the summer, and with people returning from holiday destinations in neighbouring countries, together with some super-spreading events (e.g. in the German meat industry), COVID-19 rates increased again [57] (Figure 4). However, despite the cold season approaching, no vaccine available, and herd immunity still far off, public discussion and political action were surprisingly centred around further relaxing control measures. At the same time, large-scale social gatherings and private parties continued. All this happened despite early warnings of German experts about the rapidity of a second wave in case of excessive relaxing of control measures [58]. By October, COVID-19 cases were again increasing exponentially (Figure 3), followed by a rapid exhaustion of the capacity of the local health departments (estimated to be reached at a mean incidence of >50 cases per 100,000 population for a seven-day period) [57]. As a consequence, contacts of cases could no longer be effectively traced [59]. Moreover, the testing strategy had to be adapted as it was no longer possible to test all people with symptoms and all contacts [57]. This resulted in largely uncontrolled transmission in most parts of the country [57].

Due to the rapid development of this second epidemic wave, and in view of even more serious developments in neighbouring European countries, another, albeit rather moderate, lockdown was imposed at the beginning of November 2020 [16]. It put emphasis on physical distancing wherever possible, on mandatory face masking in all public indoor as well as crowded outdoor places, and on hygiene measures including frequently ventilating indoor places, together with the use of a corona warning app. In addition, private and public social gatherings were banned; bars, restaurants, theatres, gyms, and hotels were closed, but – as opposed to the first lockdown – most business as well as preschools and schools remained open [60]. The German corona warning app puts emphasis on data protection; it does not transmit personal information to the health departments and has been considered a ‘toothless tiger’. Following established mechanisms since the first lockdown, the German government again mobilized billions of Euros to support individuals and companies economically affected by lockdown measures. However, in contrast to the first wave, there was no longer a consensus in German society about which epidemic control measures are justified and how much power the executive should be granted. This deterioration of a consensus was reflected in highly visible weekly rallies against prevention measures in German cities, by aggressive outbursts in social media, and even an arson attack against the Robert Koch Institute.

By early December 2020, the impact of the second lockdown had been disappointing. Although the exponential increase of case numbers had stopped, the number of daily reported infections continued to increase, while the effective reproduction number (R) remained around 1 (Figure 4) [57]. Moreover, an increasing number of outbreaks were seen again in nursing homes. COVID-19 cases treated in intensive care units reached an unprecedented high, and daily death rates exceeded 500 [57]. Clinical services were at the brink of becoming overwhelmed. As a consequence, a stricter lockdown started in mid-December. Germany – which has been praised for its comparably effective public health response during the first wave of the epidemic – did not succeed in preventing a second wave [7,16,61]. By December 21, Germany had reported a total of 1.53 million confirmed cases and a total of 26,427 deaths (CFR = 1.7%) [5].

## Discussion

The COVID-19 pandemic so far has caused a heavy disease burden, in particular in the WHO American Region and the WHO European Region [5,15]. The USA, the country with the highest case and death count globally, stands out for a lack of national leadership and a patchwork of responses by state and local governments but perhaps most detrimental is the division of the society along partisan lines [62]. In Latin America, a combination of failure of national leadership (e.g. Brazil), political instability, fragile health systems, and pervasive inequality is now resulting in a syndemic of COVID-19 with non-communicable diseases and a corresponding high disease burden [63].

In the initial absence of effective therapies and vaccines, NPIs were key to controlling the pandemic. Physical distancing, travel restrictions, the use of face masks and eye protection, and in particular the intensity of PCR testing have been associated with lower SARS-CoV-2 infection rates [14,64]. Large-scale lockdowns have reduced community transmission during initial outbreaks and until sufficient preparedness of the health system has been achieved, as well as during further waves of the pandemic; however, the effects usually become visible only after a delay of 1–3 weeks and depend on the intensity and combination of measures taken [20,65,66]. The reproduction number (R) is one important parameter which helps countries to monitor the effects of chosen interventions and needs to be brought below 1 to control outbreaks; but this can take a long time if infection numbers are already high [66]. As the basic reproduction number (R0) for SARS-CoV-2 is estimated to be between 2 and 3, control measures must reduce R between 50% and 67% to bring it below 1, which requires a combination of appropriate interventions to be employed for a prolonged period of time [13,14].

While about 12% of the world population is living in the WHO European Region, which accounts for about 31% of globally reported COVID-19 cases and 30% of corresponding deaths, about one quarter of the world population is living in the WHO Western Pacific Region, which accounts only for about 1% of globally reported COVID-19 cases and 1% of corresponding deaths [15]. This major difference becomes even more obvious when comparing the cumulative number of cases and deaths in countries of the west and the east, supporting the superiority of suppression compared to mitigation strategies (Table 2). Moreover, the high death rate of Sweden (e.g. in comparison to Norway) shows that a country that is following a herd immunity strategy is paying a high price for it [23]. The number of reported cases as well as the CFR depend on the intensity of testing and the characteristics of populations tested in respective countries. The very low CFR in Singapore (Table 2) is thus explained not only by the effective containment program in this city state, but also by mass testing of the population of young (usually 20–30 years old) migrant workers, who were living in crowded dormitories where SARS-CoV-2 could spread easily [11,12]. In contrast, the high CFR in China is likely the result of a certain degree of underreporting, combined with an overwhelmed health system in Wuhan during the first epidemic wave.

**Table 2.** Cumulative number of COVID-19 cases and deaths, case fatality rate (CFR), and deaths per 100,000 population in selected countries of the WHO European and the Western Pacific Region, as of 21 December 2020 https://coronavirus.jhu.edu/data/mortality

Country	Cases	Deaths	CFR	Deaths/100,000 population
*Europe*				
Belgium	625,930	18,626	3.0%	163
Italy	1,953,185	68,799	3.5%	114
Spain	1,797,236	48,926	2.7%	105
UK	2,046,161	67,503	3.3%	102
France	2,529,756	60,665	2.4%	91
Sweden	367,120	7,994	2.2%	78
Austria	338,854	5,351	1.6%	60
Russia	2,821,125	50,242	1.8%	35
Germany	1,514,962	26,400	1.7%	32
Norway	43,905	404	0.9%	8
*Western Pacific*				
Philippines	459,789	8,947	1.9%	8
Australia	28,198	908	3.2%	4
Japan	199,270	2,784	1.4%	2
Malaysia	93,309	437	0.5%	1.4
South Korea	50,591	698	1.4%	1.4
New Zealand	2,121	25	1.2%	0.5
Singapore	58,422	29	0.0%	0.5
China	95,050	4,764	5.0%	0.3
Thailand	4,907	60	1.2%	0.1
Vietnam	1,413	35	2.5%	0.04

There is an ongoing debate on the likely reasons for these large differences in the development of the pandemic in countries of the eastern and the western hemisphere, with a number of potential explanations [7,16,17,61,67]: (1) The pandemic has been considered from the beginning to be more dangerous in some of the eastern countries, probably due to previous experiences with the SARS outbreak in neighbouring China, and with the MERS outbreak in South Korea [1]. (2) This has resulted in a classification of SARS-CoV-2 in the highest WHO risk category (group 4, together with Ebola and variola viruses). As a consequence, countries in WHO Western Pacific established early aggressive control measures, while western countries classified SARS-CoV-2 only in the risk group 2 or 3, as they considered COVID-19 as an only moderately dangerous virus [68]. (3) In contrast to China and its neighbours, where the general management of cases and contacts took place mandatorily in hospitals or special public buildings, in western countries only severe cases were placed in hospitals while the majority of cases with mild symptoms as well as asymptomatic contact persons were asked to quarantine at home, often without appropriate monitoring and control measures [7,20,31]. (4) Countries of the WHO Western Pacific Region usually employed early, effective TTT interventions for both backward- and forward tracking, which allowed them to keep the number of COVID-19 cases at very low levels, and at the same time to sustain a nearly normal social and economic life [20,21,31,61,67]. (5) These countries (frequently island countries) implemented stricter travel restrictions compared to European countries [7,20]. (6) Countries in the WHO Western Pacific Region have potentially more uniform populations, and the degree of conformity may play a major role regarding compliance with painful interventions to stop outbreaks; This is supported by traditions of social distancing and the use of face masks during episodes of respiratory disease [16,67].

While countries in Europe and the Americas are now waiting for the vaccines, China has demonstrated that SARS-CoV-2 elimination is feasible even in a 1.4 billion population country [7,16,31]. Similarly, New Zealand has opted for elimination early on and has been largely successful with this strategy [22]. Being an island country with means to control immigration and thus the importation of the virus clearly has advantages. However, disease elimination is a difficult task which requires well-functioning surveillance and response mechanisms, political will and sufficient funds [69]. Interestingly, Australia – which has not officially put in place an elimination strategy – has achieved interruption of transmission through comprehensive public health measures including a major lockdown followed by intense TTT interventions in response to a second wave of the epidemic in July and August in the state of Victoria. In Japan, backward tracing (cluster tracing) seems to have been among the key interventions to keep SARS-CoV-2 infection rates low up to now; as in a given epidemic only a small number of individuals (20%) are responsible for the majority of SARS-CoV-2 infections (80%), searching for the initial clusters with TTT interventions appears to be more efficient compared to only forward tracing of many uninfected persons [21,70]. The Japanese ‘3 C’ campaign (avoid closed spaces with poor ventilation, crowded places, and close-contact settings) started in March 2020 already, and was rapidly propagated by the media; but the tracing app ‘CoCoA’ which was introduced later has not yet been successful [70].

Many other countries in the WHO Western Pacific Region were able to keep COVID-19 transmission low compared to Western countries. Only the Philippines and Indonesia appear to be outliers [5]. More intense control measures, in particular extensive TTT interventions and early aggressive lockdowns in case of outbreaks, have certainly contributed to the approximately 100-fold lower reported mortality rate (Table 2) [71]. In addition, strongly enforced travel restrictions were regularly employed, including mandatory 14-day state-supervised quarantine, supplemented by SARS-CoV-2 testing procedures, for everybody entering a country [20,67]. In China, this has allowed the economy to recover in the second and third quarter of 2020, and the social life to return to normal [20,31,67]. However, China is a profoundly different society with different priorities and much less emphasis on personal freedoms compared to Western societies. But less authoritarian nations like New Zealand, Australia, South Korea, and Thailand have also managed to minimise transmission. Social cohesion, which refers to the extent of connectedness and solidarity among groups in society, appears to be an important precondition for successful COVID-19 control programs. This concept links together individual freedom and social justice, economic efficiency and the fair sharing of resources, and pluralism and common rules for resolving all conflicts [72]. In Thailand, fissures in the social cohesion have become apparent in the second half of 2020, yet there remains a strong consensus and conformity with COVID-19 preventive measures. In contrast to rallies of people opposing COVID-19 control measures in Germany, none of the demonstrations in Bangkok in 2020 was directed against the disease preventive measures. Moreover, in Germany, as in other western countries, it appears that one important precondition for a successful epidemic response is missing – public willingness to sacrifice privacy for public health [67,73].

To control the COVID-19 pandemic globally, an epidemiologically informed, evidence-based public health response has been rightly called for, with the essential components of surveillance, outbreak investigation, TTT interventions, measures to reduce and mitigate transmission in public facilities and in the community, and research [17,61,74]. It needs to be embedded into comprehensive, resilient and publicly financed health systems providing universal health coverage, in order to be prepared for the next great pandemic [75,76].

In conclusion, social cohesion and broad conformity with early aggressive disease prevention measures are crucial preconditions for an effective control of COVID-19. China, as well as other countries in the WHO Western Pacific Region, have managed to eliminate COVID-19 or at least to sustain very low case numbers. Germany has controlled the epidemic well during the first wave but then failed to re-tighten the restrictions when cases started to increase again, resulting in a more pronounced second epidemic wave. Germany as well as other Western countries should learn from the WHO Western Pacific Region that only an early implementation of intense control measures, which keep SARS-CoV-2 numbers at a very low level, can achieve near normal social and economic life, before a successful roll-out of safe and effective vaccines will hopefully change the picture.

## Paper context

We critically examined policies and practices of COVID-19 control in China and Germany in view of the very different epidemiological development in the WHO Western Pacific and the WHO European Region. We found that China and neighbouring countries have implemented very intense control measures, which kept infection numbers low from the beginning. In sharp contrast to western countries, this has allowed them to sustain a near normal social and economic life.
